# Recent Advancements of Polymeric Membranes in Anion Exchange Membrane Water Electrolyzer (AEMWE): A Critical Review

**DOI:** 10.3390/polym15092144

**Published:** 2023-04-30

**Authors:** Rajangam Vinodh, Shankara Sharanappa Kalanur, Sadesh Kumar Natarajan, Bruno G. Pollet

**Affiliations:** Green Hydrogen Lab (GH2Lab), Institute for Hydrogen Research (IHR), Université du Québec à Trois-Rivières (UQTR), 3351 Boulevard des Forges, Trois-Rivières, QC G9A 5H7, Canada; shankara.kalanur@uqtr.ca (S.S.K.); sadeshkumar.natarajan@uqtr.ca (S.K.N.)

**Keywords:** electrolyzer, AEMWE, anion exchange membrane, anionic conductivity, oxygen and hydrogen evolution reaction

## Abstract

Water electrolysis coupled with renewable energy is one of the principal methods for producing green hydrogen (or renewable hydrogen). Among the different electrolysis technologies, the evolving anion exchange membrane water electrolysis (AEMWE) shows the utmost promise for the manufacture of green hydrogen in an inexpensive way. In the present review, we highlight the most current and noteworthy achievements of AEMWE, which include the advancements in increasing the polymer anionic conductivity, understanding the mechanism of degradation of AEM, and the design of the electrocatalyst. The important issues affecting the AEMWE behaviour are highlighted, and future constraints and openings are also discussed. Furthermore, this review provides strategies for producing dynamic and robust AEMWE electrocatalysts.

## 1. Introduction

The growing energy needs around the globe, the exponential increase in green-house gas emission and the gradual depletion of fossil fuels is a major concern [1,2]. Hydrogen (H_2_), as a potential energy vector, exhibits a crucial part in the net zero car-bon emissions domain owing to its numerous benefits, such as high energy content, sustainability, and non-toxicity [3,4]. The usage of H_2_ alleviates the ecological effects that arise with carbon-derived fuels. However, different from non-renewable energy, H_2_ used as a form of fuel does not exist in the environment [5]. More than 95% of H_2_ gas has been manufactured by high temperature reforming methods, a well-established technique consuming fossil fuels that comprise predominantly methane (SMR process—steam methane reforming) [6,7,8]. Nevertheless, the reforming of fossil fuels releases a huge quantity of carbon dioxide (CO_2_), and the H_2_ manufactured by this method is termed grey H_2_ [9]. As a way to make green H_2_, where the hydrogen is produced by naturally available resources in the absence of carbon dioxide release, water electrolysis (WE) power-driven by natural resources is evolving as a maintainable and profitable hydrogen manufacturing technique.

**Scheme 1:**
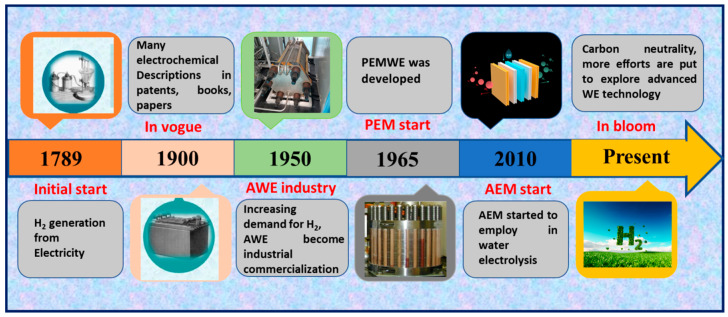
Schematic timeline illustration for developing WE technology.

Before we discuss the state-of-the-art technologies, the history of WE is briefly presented as depicted in Scheme 1. As early as 1789, Adriaan et al. cast off an electrostatic generator to decompose water into H_2_ and oxygen (O_2_), primarily understanding the generation of hydrogen from electricity [10]. Subsequently, water-splitting reactions and electrolysis reactors were extensively studied at the end of the 19th century due to the founding of electrochemistry. Starting from the middle of the 20th century, with the growing demand for hydrogen in the chemical manufacturing sector, AWE underwent industrial commercialization; however, it was only exploited for a brief period and then rapidly exiled by the petrochemical industry. Because of the low market prerequisite, WE technology was quiet and gradually established for decades. In 1965, the new epoch of PEM-based water electrolysis commenced [11]. With the renewed interest in using renewable energies at the beginning of the 21st century, PEM became popular owing to its distinct merits of directly producing high-purity and pressured hydrogen. At present, AEM has started to be employed in WE. Currently, progressive WE technologies are competing in the same race to achieve carbon neutrality and a sustainable future. 

### 1.1. Water Electrolysis

**Figure 1:**
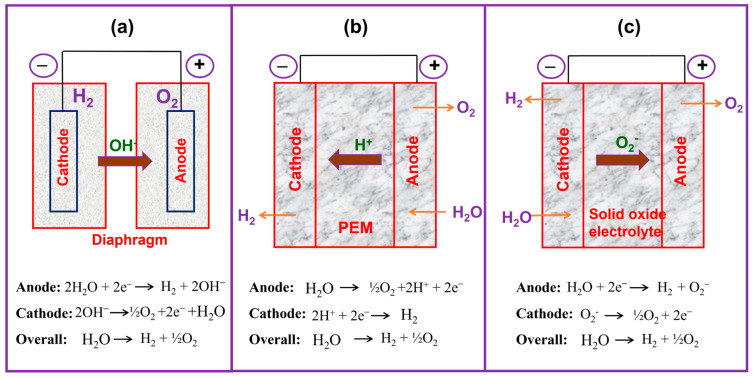
Comparative pictures of the different electrolyzer techniques: (**a**) AWE, (**b**) PEMFC, and (**c**) SOE.

WE is one of the notably efficient techniques for the production of H_2_ since it requires sustainable water (H_2_O) and makes only pure oxygen (O_2_) as an additional product. Moreover, the water electrolysis method exploits the direct current (DC) from renewable energy resources, such as wind, biomass, and solar. However, currently, only about 4% of H_2_ can be acquired by WE technology, primarily owing to economic concerns [12,13]. This value is anticipated to increase in the subsequent years, though it will enhance the use of sustainable power (wind, solar, and nuclear). Moreover, WE having threatening benefits such as high cell efficacy and a superior rate of H_2_ production with extraordinary cleanliness are added advantages for its further transformation into electrical energy by low-temperature fuel cells (LTFCs) [14]. During the water electrolysis process, an H_2_O molecule is the reactant; it is separated into H_2_ and O_2_ in the impact of current. WE can be mainly categorized into four different varieties depending on their electrolytes, operational temperature, operating circumstances, and ionic moieties (H^+^, OH^−^, and O^2−^); nevertheless, the operating mechanisms are the same in all types. The different water electrolysis techniques are (i) alkaline water electrolysis (AWE) [15,16,17], (ii) H^+^ (proton) electrolyte membrane water electrolysis (PEMWE) [18,19], (iii) anion exchange membrane water electrolysis (AEMWE) [20,21], and (iv) solid oxide electrolysis (SOE) [22,23]. The pictorial representation of the AWE, PEMWE, and SOE techniques is given in Figure 1. 

Furthermore, recently, the employment of bipolar membranes (BPMs) has been anticipated in dual-electrolyte water electrolysis schemes, as it gives diverse advantages that could overcome the drawbacks of traditional single-electrolyte water electrolysis [24,25]. A BPM embraces a cation-selective layer with fixed negative charges penetrable to only cations and an anion exchange layer with positive charges that allows the migration of only anions. When BPM is combined into the water electrolyzer system, it associates with the rewards of both PEM and AEM electrolyzers by allowing HERs to favorably take place in the acidic cathode and OERs in the basic electrode, respectively, thereby reducing the overall overpotential of the cell [26]. This also reduces severe catalytic selectivity for HERs and OERs, which is defined by a typical single pH environment water electrolysis system [27,28].

### 1.2. Different Types of Water Electrolyzers

#### 1.2.1. AWE

H_2_ produced from the AWE technique (Figure 1a) is a well-matured method up to the commercial level, reaching 1000 kilowatts, and the occurrence was first presented by Troostwijk and Diemann in 1789 [29,30]. In the AWE process, first, on the cathode path, two molecules of alkaline KOH/NaOH are reduced to one hydrogen molecule, and two hydroxyl (OH^−^) ions are formed. The formed H_2_ is eliminated from the cathode surface to reunite in a gaseous form and the OH^−^ ion is transferred through the cathode and anode in the effect of the electrical circuit. A porous diaphragm at the anode discharges half a molecule of O_2_ and one molecule of H_2_O. Oxygen reunites on the surface of the electrode and escapes as H_2_. AWE works at lower temperatures between 30 and 80 °C with KOH/NaOH solution as the electrolyte; the molarity of the electrolyte is approximately 20% to 30% [31,32]. Generally, a porous asbestos diaphragm and nickel-based components are used as the electrode material in AWE. The diaphragm is positioned at the center of the electrolysis unit, and it splits the cathode and the anode and divides the formed gases from their corresponding electrodes and avoids the mingling of the produced gases during the process of electrolysis. Nevertheless, AWE has destructive issues such as restricted current densities (<400 mA cm^−2^), inferior working pressure, poor power efficacy, and the inefficacy to produce high-purity and pressured gas products [33]. Generally, the formed gas consists of alkaline liquid and water vapor, which require elimination by supporting equipment. In addition, it is hard for AWE to start up rapidly or change the load to alter the speed of H_2_ generation rapidly, so it is poorly suited for renewable energy generation (for example, wind power generation, photovoltaic power generation). 

#### 1.2.2. PEMWE

PEMWEs consist of a proton electrolyte membrane (PEM) and an ionomer (e.g., Nafion^®^) in the electrode design; it permits the chamber operation with the absence of a circulating liquid electrolyte (Figure 1b). In their arrangement, both the cathode and anode electrodes are in physical interaction with a proton electrolyte membrane (non-porous), occurring in condensed cell configurations (zero-gap arrangement), and it permits the working of PEMWEs at approximately 2 A cm^−2^ of current density (C_D_) with an energy effectiveness of 74% [34]. Further, the non-porous membrane of proton exchange membrane water electrolysis permits variance in the operating pressure and yields a high-pressure H_2_ at the cathode and atmospheric oxygen pressure at the anode. Such differing pressure setups could decrease the requirement for the next step of mechanical constricting to pressurize for H_2_ stowage. Despite these benefits, the expensive electrocatalysts (IrO_2_ and Pt) required and the corrosion-resilient current collectors and divider plates in acidic atmospheres may become drawbacks for large systems, as the cell stack becomes a superior donor to the entire scheme price [35]. Both PEMWEs and AWEs are deliberately developed methodologies and have been organized at a marketable measure reliant on the precise necessities of their use. 

#### 1.2.3. SOE

In the 1980s, the SOE technique was first developed by Donitz and Erdle [36]. SOE has attracted considerable attention among researchers owing to it changing electrical energy into chemical energy, along with making ultra-pure H_2_ with superior productivity [37,38]. The SOE works at high pressure and high temperatures between 500 and 850 °C and outputs the water in steam form. The SOE method usually applies the O^2−^ conductors which are typically made from nickel/yttria-stabilized zirconia [39] and its schematic is presented in Figure 1c. One of the key features of the SOE method is that it can operate at a higher temperature, which is one of the beneficial parameters when compared with low-temperature WE techniques. However, the solid oxide electrolysis methods have a few concerns associated with the deficiency of durability and deprivation, which have to be addressed prior to large-scale production and industrialization [40,41]. 

### 1.3. AEMWE and Its Working Principle

**Figure 2:**
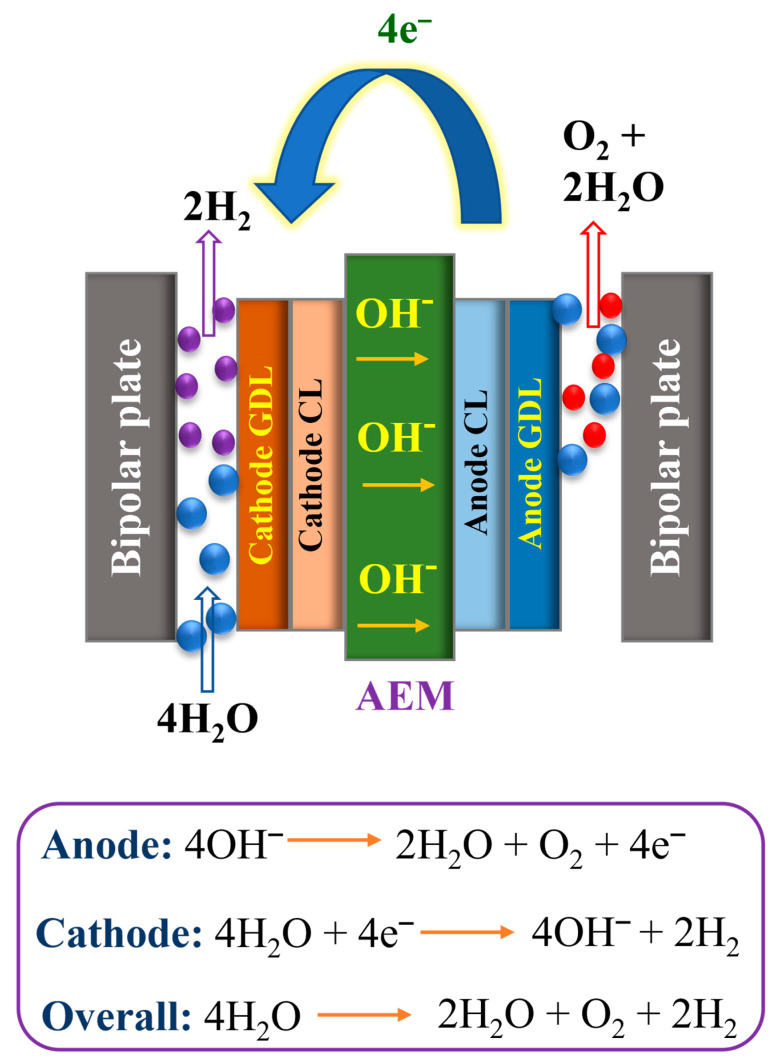
Schematic representation of the AEMWE.

The main constituents of the AEMWE cell comprise an anion exchange membrane, gas diffusion layers, electrocatalysts, and current collectors. The simple working mechanism of the AEMWE system is illustrated in Figure 2. The total reaction of AEMWEs comprises O_2_ evolution reaction (OER) and H_2_ evolution reaction (HER). H_2_O or a basic liquid electrolyte is distributed via the cathode, where H_2_O is reduced to H_2_ and OH^−^ ions by adding four electrons from the anode (4H_2_O + 4e^−^ → 2H_2_ + 4OH^−^). The OH^−^ ions diffuse via the anion exchange membrane to the anode, whereas the electrons are conveyed to the cathode direction by the exterior circuit. At the anode side, the OH^−^ ions reunite as O_2_ and H_2_O and yield four electrons (4OH^−^ → O_2_ + 2H_2_O + 4e^−^). The O_2_ and H_2_ form sparkles on the surface of the OER and HER electrocatalysts, respectively. According to the equation in Figure 2, it is noteworthy to mention that the water detachment occurs at the cathode during the HER and it is related to a comparatively huge overpotential. Indeed, although numerous HER catalysts exhibit excellent behavior in an acid medium, HER performance in an alkaline medium is low owing to the absence of appropriate dynamic spots to adsorb OH and expedite the H_2_O detachment [42]. Furthermore, OER is accompanied by comparatively large overpotential and the OER reaction kinetics are quite vague. The theoretically reversible voltage under typical environments is 1.23 V, as determined by the free Gibbs energy (G) of water splitting [43]. However, in general, the working voltage of AEMWE devices is very high, which is primarily due to the kinetic energy and iR drop (ohmic resistance). To decrease the overpotential, anion exchange membranes with good ion conductivity and electrodes with high catalytic behavior must be developed towards more efficient AEMWE units. Like PEMWEs, the zero-gap arrangement of AEMWEs uses a non-porous membrane, which can yield H_2_ at a high rate and reduce the need for mechanical hydrogen compression for stowage [44]. It is noteworthy that AEMWEs utilize both AWEs (PGM-free catalyst) and PEMWEs (zero-gap arrangement and non-porous membrane). Fascinatingly, different from PEMWEs that use only polymer electrolytes, several AEMWEs frequently use liquid electrolytes of KOH or K_2_CO_3_ solutions in addition to polymer electrolytes. The current work showed that the addition of liquid electrolytes not only decreases the iR resistance of the membrane and catalyst layer but also enhances the reaction mechanism [45]. With the addition of liquid electrolytes to the electrolysis cell, the local pH at the electrode and catalyst interface enhances and an extra electrochemical interface is created as well. Interest in H_2_ generation through AEMWEs is increasing because of the reduced cost of electrocatalysts and computer hardware, appropriate zero-gap arrangements, and differences in working pressure. The main characteristics and advantages/disadvantages of different electrolyzer techniques are condensed in Table 1 and Table 2, respectively.

## 2. Anion Exchange Membrane

The anion exchange membrane is the heart of AEMWE systems, which can considerably enhance the electrode strategy and shorten the gas organization [46,47]. Certainly, the AEM can separate the formed H_2_ and O_2_ gases, instantaneously permitting the passage of OH^−^ ions and H_2_O molecules to balance the charges and reactants required for the OER and the HER in the anode and cathode electrodes, respectively. AEM’s compact structure is built with polymer supports and anion exchange functional moieties. The functional moieties are typically the ammonium, diammonium, and phosphonium group [48], which can be connected to the key chain or the extended branched chains of the polymer support. The modification of these moieties can enhance chemical stability. Mostly nitrogen-based groups have been examined as the functional moieties, which include quaternary ammonium [49], heterocyclic systems [50], guanidinium systems [51], phosphonium-based systems [52], sulphonium types [53], and metal-based systems [54]. As for the backbone of AEM, the mechanical strength and ionic conductivity of poly (arylene ethers) [55], poly phenylenes [56], polyethylen [57], poly styrene [58], and poly (vinyl alcohol) [59] have also been extensively explored. When matched with H^+^ transmission in the polymer electrolyte membrane, the transmission of OH^−^ ions in the anion exchange membrane is much gentler [60]. This might be due to the lesser inherent movement of bigger ions in mass and volume. When the polymer electrolyte grips water and swells, the hydrophilic sections and hydrophobic sections are dissociated, and the OH^−^ ions are transported into the hydrophilic networks. The transportation of OH^−^ approaches could be designated as Grotthus, vehicular, and surface mechanisms [61]. Alternatively, the small acceptance of PEM to metal ions confines its industrialization, whereas the AEM in basic environments allows a greater selection of non-noble metal catalysts. Because of its vital role in harmonizing the charge and separating the produced gases, AEM must encounter the subsequent practical necessities: (i) excellent OH^−^ ion conductivity (at least 100 mS cm^−1^), (ii) decent alkaline durability and thermal strength, (iii) low inflammation rate, and (iv) exceptional mechanical characteristics. In this mini review, we primarily focus on the recently reported anion exchange membranes in AEMWE applications. 

Various state-of-the-art AEM products have been commercialized, including Fumasep^®^ FAA3, Tokuyama A201, Aemion^TM^, Sustainion^®^ 37–50, Durion TM1, and PiperION [62,63,64,65,66,67]. Commonly, they can be unswervingly employed as an alkaline electrolyzer after a possible pretreatment with potassium hydroxide. Some outstanding AEMWE behavior has been described for these commercially available anion exchange membranes. Nevertheless, the commercial AEMs still cannot comprehend long-term durability at 1 A cm^−2^ for >3000 h, which is primarily due to their chemical stability. In a highly alkaline atmosphere, the OH^−^ ion tends to attack the backbone and functional groups of AEMs by different mechanisms such as nucleophilic substitution reactions and Hofmann elimination, leading to chain scission, deactivation, and degradation. Thus, recent AEM research is more fixated on emerging stable backbones and attaining a balance between the hydroxyl ion conductivity and functional group stability.

### 2.1. Recent Reports of Anion Exchange Membrane for AEMWEs

#### 2.1.1. Quaternary Ammonium-Based Anion Exchange Membrane

**Figure 3:**
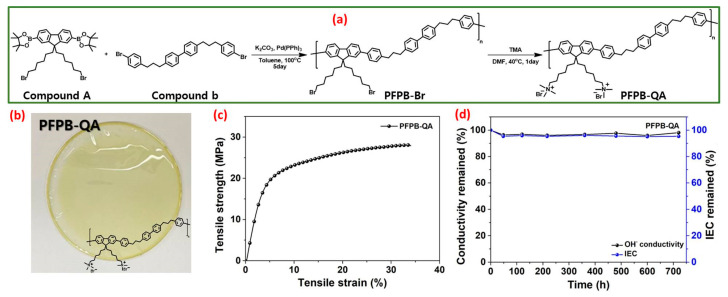
(**a**) Schematic illustration of the PFPB-QA synthesis route; (**b**) digital image of PFPB-QA membrane; (**c**) mechanical characteristics of PFPB-QA; (**d**) ion exchange capacity and conductivity as a function of time. Reproduced with permission from [68]. Copyright 2023 Elsevier.

Very recently, Haeryang Lim et al. synthesized a polyfluorene-based quaternary ammonium-based poly [9,9′-bis (6-bromohexyl) fluorene]-co-[4,4′-bis((4-phenyl) propyl) biphenyl)] (PFPB-QA) polymer with an alkyl spacer and an anchored cation that could be employed as an anion exchange membrane and an anion exchange ionomer [68]. The synthetic scheme of PFPB-QA is depicted in Figure 3a. PFPB-Br was successfully prepared from compound A (2,7-bis (4,4′,5,5′-tetramethyl-1,3,2-dioxaborolan-2-yl)-9,9′-(6-bromohexyl) fluorine) and compound B (4,4′-bis(3-(4-bromophenyl) propyl) biphenyl) via the Suzuki coupling reaction. Subsequently, the quaternization reaction was carried out in PFPB-Br via the Menshutkin reaction with trimethyl amine and dimethyl formamide at 40 °C for 24 h to achieve PFPB-QA. The digital image of the quaternized PFPB AEM is depicted in the inset of Figure 3b, and it was bendable and hard. The tensile strength (T_S_) and hydrolytic stability of anion exchange membranes are crucial aspects for prolonged intrinsic physical and chemical stability in AEMWEs. Therefore, the tensile strength and stretching behavior, that is, elongation at break (E_B_), of the quaternized PFPB AEM were examined at 50% relative humidity. The hydroxyl form of the quaternized PFPB membrane showed a remarkable T_S_ of 28 MPa and 33.9% for E_B_, even with a small molar mass. The excellent characteristics of the quaternized PFPB AEM are ascribed to the decreased glass transition temperature from the alkyl spacer in the main skeleton of the polymer; it permits the creation of a thicker architecture while molding the anion exchange membrane (Figure 3c). The hydrolytic durability of the quaternized PFPB AEM is observed by OH^−^ ion conductivity and ion exchange capacity after the membrane specimens are immersed in 1 M potassium hydroxide solution for 30 days at 80 °C (Figure 3d). In the observation cycle, no color change was observed in the PFPB-QA AEM, and the AEM was not damaged. During the alkaline stability test, the quaternized PFPB AEM retained OH^−^ conductivity of 97% when associated with the original number over 30 days. Furthermore, the drop in ion exchange capacity value was 95% associated with the original value after 30 days, which remained reliable with the OH^−^ ionic conductivity. Moreover, the synthesized PFPB-QA AEM displayed an outstanding OH^−^ conductivity of 0.122 S cm^−1^ at 80 °C and exceptional alkaline durability in 1 M KOH at 80 °C for 30 days. Furthermore, the water electrolyzer exploiting quaternary ammonium-based PFPB displays a maximum current density of 1.53 A cm^−2^ at 2 V in 1 M KOH at 70 °C.

C. Simari et al. synthesized quaternized polysulfone (qPSU) AEM using environmentally friendly and low-cost polysulfone as the starting material [69]. The qPSU AEM showed remarkable alkaline stability, TS and EB resulting from a nano-phase separation between hydrophilic and hydrophobic territories in such electrolytes. The final features permit the creation of long penetrating ionic moieties in the quaternized polysulfone membrane, producing a well-organized passageway for effective OH^−^ ion transmission. The prepared qPSU AEM showed an exceptional C_D_ of 4.2 A cm^−2^ at 2.2 V in an AEMWE unit working at 90 °C. Furthermore, the long-term durability of the AEMWE study is not present and it will be the objective of an upcoming study. 

Min Suc Cha et al. reported quaternized poly (carbazole)-derived AEM (QPC-TMA) with remarkable ionic conductivity and stability behavior for water electrolysis applications [70]. The prepared QPC-TMA anion exchange membrane demonstrates outstanding durability and displayed a superior performance of 3.5 A cm^−2^ at 1.9 V in an AEMWE unit. 

#### 2.1.2. Cross-Linked Anion Exchange Membrane

To address the “compromise” between anionic conduction behavior and durability of AEM, Maolin Guo et al. fabricated a sequence of cross-linked membranes by using polybenzimidazole (PBI) with norbornene (Nb) (cPBI-Nb) as the main skeleton and the cross-linked assembly by assuming click chemistry between the vinyl group and thiol [71]. In the meantime, the hydrophilic characteristics of the dithiol cross-linker were controlled to discover the influence of tiny-phase separation structure and OH^−^ ionic conduction. Thus, the AEMs with hydrophilic cross-linked morphology (PcPBI-Nb-C2) not only had deceptive tiny-phase separation structure and extraordinary hydroxyl conduction of 0.105 S cm^−1^ at 80 °C but they also displayed enhanced power-driven characteristics and dimensional stability. Furthermore, the AEMWEs employing PcPBI-Nb-C2 as membranes attained a C_D_ of 368 mA cm^−2^ at 2.1 V, and the stability study was operated at 150 mA cm^−2^ for 500 min below 60 °C.

Wangting Lu et al. reported a bicomponent anion exchange membrane in the form of poly (vinyl benzyl chloride) (PVBC) cross-linked with PBI, and quaternization by *N*-methylpiperidine (NMPD) was carried out [72]. The anion exchange membrane with a PBI/PVBC weight ratio of 1:1 (PBI1-PVBC1-NMPD/OH) offered OH^−^ ion conductivity greater than 80 mS cm^−1^ at 80 °C. Additionally, the supplementary influence of polybenzimidazole and interconnected architecture provides the AEM with decent tensile strength and almost temperature-independent water acceptance (<50%) and inflammation ratio (~10%). After immersing in 1 M KOH at 60 °C for 336 h, ~80% of OH^−^ ionic conductivity of PBI1-PVBC1-NMPD/OH was retained. Furthermore, the authors demonstrated the viability of PBI1-PVBC1-NMPD/OH employed in AEMWEs. 

#### 2.1.3. Piperidinium Functionalized Anion Exchange Membrane

Ziqi Xu et al. reported an anion exchange membrane with a tri-block co-polymer of styrene–ethylene–butylene–styrene (SEBS) as its main skeleton and piperidinium functionalized stretchable ethylene oxide spacer assembly as its branched chain (SEBS-P2O6) [73]. The SEBS-P2O6 AEM achieved a maximum ionic conductivity of 20.8 mS cm^−1^ at ambient temperature. The SEBS-P206 was employed in a single-cell AEM electrolysis unit with a PGM catalyst. The current densities of 275 mA cm^−2^ and 680 mA cm^−2^ at 60 °C and 2 V cell potential were attained in ultra-pure water and 0.1 M KOH, respectively.

#### 2.1.4. Composite and Blended Anion Exchange Membrane

Sommayyeh Rakhshani et al. prepared a composite AEM employed by triggering a viable backing structure (Celgard^®^ 3401) with a practically accessible efficient moiety (Fumion^®^ FAA-3) via a phase-inversion procedure [74]. Furthermore, the composite AEM (Celgard^®^/Fumion^®^) performance results in AEMWE cells were quite encouraging and far better than the commercially available membrane in alkaline electrolysis. 

**Figure 4:**
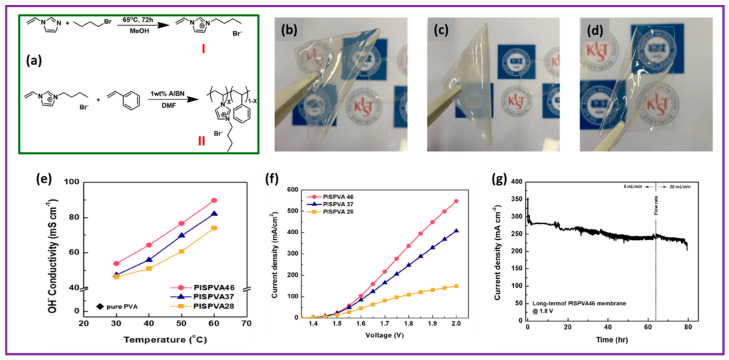
(**a**) Synthesis procedure of PIS; (**b**–**d**) photos of PISPVA46, PISPVA37, and PISPVA28 membranes, respectively; (**e**) hydroxyl ion conductivity of PISPVAx membrane with respect to temperature; (**f**) polarization curve of various PISPVAx membranes; (**g**) durability performance of the PISPVA46 membrane. Reproduced with permission from [75]. Copyright 2020 Elsevier.

Hyun Jin Park et al. demonstrated blended membranes composed of a sequence of N3-butyl-substituted imidazolium-based poly(1-vinyl-3-imidazoleco-styrene) (PIS), synthesized via the radical polymerization method, and PVA employed as anion exchange membrane for AEMWE applications [75]. The synthesis procedure is depicted in Figure 4a and a brief explanation is as follows. It involves a two-step preparation process. First, ionic liquid of C_4_VIBr (1-butyl-3-vinylimidazolium bromide) was prepared by refluxing 1-vinylimidazole and 1-bromobutane in methanol at 65 °C for 72 h. The obtained reactant (C_4_VIBr) was desiccated for 24 h in a vacuum atmosphere at 60 °C to eliminate the impurities exhibited in the form of unreacted C_4_VIBr. A transparent, purple-colored liquid was attained. Second, PIS was prepared via free-radical polymerization. C_4_VIBr was mixed in dimethyl formamide under an Ar environment. After thorough solubility, a styrene monomer and AIBN were added to the free-radical polymerization. The whole polymerization was maintained for 12 h at 75 °C. Then, the reaction content was poured into DI water to precipitate and was cleaned several times with copious amounts of distilled water. The final material, PIS46 (the number 46 in the formula signifies the molar ratio of C_4_VIBr and styrene unit), was desiccated at 80 °C for 24 h in a vacuum oven. By altering the molar ratio of C_4_VIBr and styrene, PIS37 and PIS28 copolymers were prepared following the aforementioned procedure as well. PVA was efficaciously united with three prepared PISs by mixing PVA and PIS in three dissimilar molar parts. All of them displayed an outstanding thin-film-forming capacity and were not soluble in water and potassium hydroxide medium. The images of the blended membranes are shown in Figure 4b–d. The ion exchange capacity, water acceptance, and OH^−^ conductivity of the PISPVA46 membrane were as high as 1.65 mmol g^−1^, 101.1%, and 0.089 S cm^−1^, respectively, in 0.5 M KOH at 60 °C. The hydroxide ion conductivities of the mixed membranes soaked in 6 M KOH with varying temperatures between 30 °C and 60 °C in 0.5 M KOH are exhibited in Figure 4e. The PISPVA46 AEM shows considerably enhanced hydroxyl ion conductivity at all the temperatures when matched with the other blended AEMs. The PISPVA46 AEM had a maximum hydroxyl ion conductivity between 53.9 and 89.7 mS cm^−1^, attributed to its extraordinary water retention characteristics. This was followed by the PISPVA37 membrane (47.4 to 82.1 mS cm^−1^) and PISPVA46 membrane (46.3 to 74.1 mS cm^−1^) owing to their poor water-absorbing characteristics. It is important to mention that PISPVA*x* AEMs exhibit high OH^−^ ionic conductivity at higher temperatures with high content of imidazole. When the KOH moieties are presented into poly (vinyl alcohol), the hydroxyl group in PVA becomes hydrolyzed, ensuing in a change in the crystalline phase of PVA into the amorphous phase [76,77,78,79]. The hydroxyl ionic conductivity of the blended membranes enhances owing to a few aspects such as (1) the jumping mechanism between co-ordinate moieties, (2) local structural recreation, and (3) the fragment gesture in the polymer at all temperatures. As the fragmental motion seems effortless in the polymer amorphous phase, the superior conductivity of the blended membranes appears to predominantly be due to the fragmental motion of the amorphous PVA after incorporating with KOH [80]. Cell voltage plots of PISPVAx blended membranes are shown in Figure 4f. The membrane electrode assembly employing PISPVA46 AEM shows a C_D_ of 547.7 mA cm^−2^ at 2.0 V, i.e., superior behavior to the PISPVA37 and PISPVA28 AEMs presenting 408.1 mA cm^−2^ and 149.0 mA cm^−2^ at 2.0 V, respectively. The PISPVA46 AEM exhibits an improved electrochemical behavior compared to the other blended AEMs, reliable with the detail that the Imidazolium-*f*-PISPVA46 membrane possesses high anionic conductivity and water retention characteristics. Long-lasting durability was explored to display the behavior change owing to the AEM deprivation in an alkaline environment. Figure 4g illustrates the long-lasting durability outcomes for the PISPVA46 AEM attained after calculating the electrolyzer behavior in 0.5 M KOH solution at 1.8 V at 60 °C. The electrolyzer behavior dropped sharply from 354.1 mA cm^−1^ to 285.6 mA cm^−1^ in the initial phase because of the modifications of the working conditions. This clearly indicates a decline in behavior within 8% from 30 min after moving to nearly 40 h and exposed steady driving outcomes. After the process was finished at 80 h, a performance drop of 21% at 224 mA cm^−1^ was noticed. 

#### 2.1.5. Morpholine Modified Anion Exchange Membrane

Simone Bonizzoni et al. synthesized AEM by a three-step method. In Step 1, the Paal–Knorr (PK) reaction between a 1,4-diketone unit and *N*-(3-aminopropyl)-morpholine incorporates a pyrrole ring into the polymer backbone [81]. In Step 2, the ternary amine of the morpholine ring is changed into the analogous tetraalkylammonium salt by alkylation with CH_3_I. Lastly, in Step 3, the iodide-based membranes are soaked in 1 M KOH solution for 2 h to interchange the anionic groups, then thoroughly cleaned numerous times with deionized water and desiccated at ambient temperature. The obtained AEMs exhibited excellent IEC values in the range between 1.48 and 2.24 meq. g^−1^. The current achieved through this AEM in water electrolysis cells is relatively small (60 mA cm^−2^ at 2.5 V) compared to other commercial water electrolyzers. Hence, the authors suggested that further optimization is required for future study. 

#### 2.1.6. Piperidinium-Based Anion Exchange Membrane

Nanjun Chen et al. synthesized poly (fluorenyl-co-aryl piperidinium) (PFAP)-derived AEM for AEMWE applications [82]. Besides having a balanced electrode scheme, PFAP-derived anion exchange membranes with a high rate of water diffusion and OH^−^ ionic conductivity are vital for high-performance AEMWE applications. Employing PGM electrocatalysts, the AEM water electrolyzer attained a current density of 7.68 A cm^−2^ at 2.0 V in a 1 M KOH anode, which beats that of ultra-modern PEMWEs (6 A cm^−2^ at 2.0 V). Non-PGM AEMWEs exhibited an outstanding C_D_ of 1.62 A cm^−2^ at 2.0 V. Notably, with and without PGM, AEMWEs functioned steadily in 0.5 A cm^−2^ C_D_ for over 1000 h at 60 °C.

Lei Liu et al. demonstrated side-chain structural engineering of poly (terphenyl piperidinium) (PTP) polymers depending on the incorporation of N-oligo (ethylene glycol) (OEG) terminal pendants, which was executed to produce the polymer crystalline and tune the local hydrophilic surroundings of organic cations, aiming to enhance the alkaline durability of anion exchange membranes while maintaining extraordinary OH^−^ ionic conductivity [83]. The brief synthesis procedure was as follows. First, the polymer of PTPip was synthesized via Friedel–Crafts alkylation of *p*-terphenyl and 4-methyl piperidone, consisting of an aryl-ether-free main skeleton and methyl piperidine functionality. Consequently, the polymer of PTP-OEG4 was synthesized via the Menshutkin reaction between PTPip and surplus bromide-*f*-OEG in DMSO at 80 °C. Exploiting the PTP-OEG4 membrane, the established AEMWE cell was evaluated at a high C_D_ of 5.9 A cm^−2^ at 2.2 V by employing Pt-Ru/C and IrO_2_ as the cathode and anode electrocatalysts, respectively. The obtained behavior is analogous to that of a proton exchange membrane water electrolysis cell consisting of a trademarked Nafion^®^ membrane and surpasses that of the Department of Energy (DOE, USA)’s state-of-the-art objective for AEMWE schemes. Prominently, the AEMWE cell based on the PTP-OEG4 AEM exhibited enhanced stability when matched with PTP-C1 AEM at a high C_D_ of 1 A cm^−2^ worked after 300 h.

Tommaso Caielli et al. successfully synthesized and characterized poly (biphenyl piperidinium)-based AEM for anion exchange membrane water electrolyzer cells [84]. The AEM attained an outstanding OH^−^ conductivity of 185 mS cm^−1^ at 80 °C with 100% RH. Moreover, nearly no degradation was observed over 360 h in 1 M KOH at 80 °C. The maximum C_D_ of 2.8 mA cm^−2^ was attained at 60 °C in a single-cell AEMWE unit. Furthermore, the unit cell studies obviously exemplified improved cell behavior compared to the commercially available AEM of PiperION™.

Xiaoming Yan et al. successfully prepared a twisted ether-free polyarylene piperidinium (QMterco-Mpi) via a facile and well-regulated acid-catalyzed polycondensation reaction for AEMWEs [85]. The OH^−^ ionic conductivity of QMter-co-Mpi AEM attains 37 mS cm^−1^ at 30 °C because well-organized ion-transporting pathways are created by means of this exceptional molecular architecture. Moreover, the QMter-co-Mpi employed by AEMWE exhibits a high C_D_ of 1064 mA cm^−2^ at 2.5 V in 1 M KOH at 50 °C. The stability study was achieved at a C_D_ of 200 mA cm^−2^ for more than 500 h and the voltage was mainly retained at 2.1 V, illustrating an outstanding water electrolyzer behavior. 

**Figure 5:**
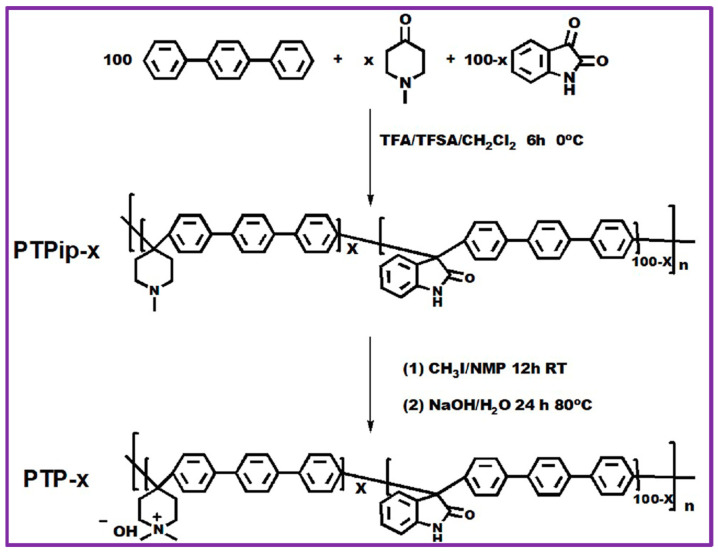
Reaction protocol of PTP-x synthesis. Reproduced with permission from [86]. Copyright 2021 Elsevier.

Xu Hu et al. described a sequence of quaternary ammonium-based poly [(terphenyl piperidinium)-co(oxindole terphenylylene)] (PTP) for an AEM water electrolyzer [86]. The schematic representation of the reaction scheme is illustrated in Figure 5. PTPip-x co-polymers were prepared via super-acid-catalyzed polymerization of *p*-terphenyl, *N*-methyl-4-piperidone, and isatin, where x designates the molar ratio of *N*-methyl-4-piperidone to isatin. In general, to synthesize the PTPip-85 copolymer, the required amounts of *p*-terphenyl, isatin, *N*-methyl-4-piperidone, and anhydrous dichloromethane were placed into a three-necked 100 mL RB flask equipped with a mechanical stirrer. Then, trifluoromethanesulfonic acid (TFSA) and trifluoroacetic acid (TFA) were slowly added to the reaction mixture at 0 °C and allowed to continue reacting for another 6 h. After the completion of the reaction, the dark green/blue-colored precipitate was decanted into an aqueous ethanol solution. Lastly, the white-colored fibrous material was precipitated, cleaned with Millipore water, and desiccated at 60 °C. Subsequently, the precursor PTPip-x copolymer was completely subjected to quaternization via Menshutkin reactions by CH_3_I to give PTP-x co-polymers. 

**Figure 6:**
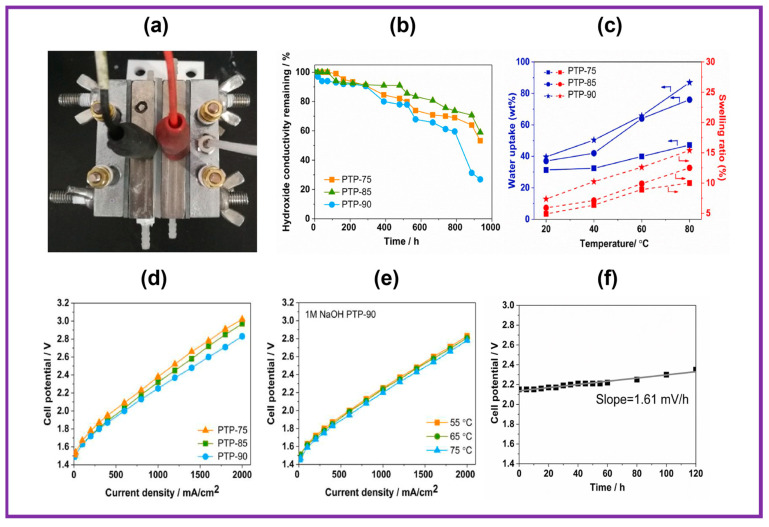
(**a**) Photo of indigenous water electrolyzer setup; (**b**) alkaline durability of PTP membrane in 1 M sodium hydroxide at 80 °C; (**c**) water absorption and swelling ratio of PTP membrane as a function of temperature; (**d**) I–V curves for the AEMWEs with PTP AEM at 55 °C; (**e**) AEMWEs with PTP-90 at various temperatures; (**f**) durability study of AEMWEs with PTP-85 at 55 °C at 400 mA cm^−2^. Reproduced with permission from [86]. Copyright 2021 Elsevier.

The indigenous electrolyzer unit is exhibited in Figure 6a. The catalyst-coated substrate (CCS) technique was employed to construct the membrane electrode assembly (MEA), employing Pt/C and IrO_2_ as the cathode and anode electrocatalysts, respectively. The water retention characteristics of the prepared PTP AEMs were plotted and are displayed in Figure 6c. As anticipated, all AEMs displayed enhanced water absorption with rising temperatures between 20 and 80 °C. Mainly, PTP-90 AEM showed the greatest ion exchange capacity value, showing the highest dependency of H_2_O absorption on temperature, with 86.9% higher water absorption at 80 °C. In addition, the H_2_O absorption of PTP-75 AEM improved by 1.5-fold at 80 °C when compared to 20 °C. The analogous pattern was observed for the swelling ratio at higher temperatures. In spite of its supreme ion exchange capacity value and high-water absorption, the PTP-90 membrane exhibited outstanding hydrolytic stability even at 80 °C, which is highly desirable for water electrolyzer applications. The alteration in OH^−^ conduction behavior was supervised at pre-determined intervals, as shown in Figure 6b. All the membranes exhibited constant damage in conductivity throughout the test interval. The AEM PTP-90 with a high IEC value displayed inferior durability in alkaline environments. Specifically, the OH^−^ conductivity of the aged PTP-90 AEM declined by 73% over 934 h of alkaline testing. In contrast, 41% of damage in OH^−^ conductivity was realized for PTP-75 membranes under similar experimental environments. The low values of IEC and water absorption might be associated with the high alkaline durability of the PTP-75 AEM. As revealed in Figure 6d, the behavior of a PTP-based alkaline water electrolyzer at 55 °C is highly dependent on the membrane characteristics. As anticipated, the AEM with high OH^−^ ionic conductivity achieved improved behavior due to its lower AEM resistance. For instance, the MEA with PTP-75 had a C_D_ of 767 mA cm^−2^ at 2.2 V at 55 °C, while a higher C_D_ of 910 mA cm^−2^ was noticed at 2.2 V for the MEA with highly conductive PTP-90 AEM. Figure 6e shows the polarization curves of the MEA with the PTP-90 membrane as a function of temperature. The C_D_ improved from 910 to 1000 mA cm^−2^ at 2.2 V of MEA with PTP-90 membrane as the temperature amplified from 55 °C to 75 °C. The durability of the electrolyzer with MEA was expected at 55 °C under a C_D_ of 400 mA cm^−2^. The change in cell voltage was monitored as depicted in Figure 6f. The cell voltage at the commencement of the stability test is higher than in the primary I–V curve due to the irreversible efficiency loss during several measurements by increasing the C_D_ from 10 to 2000 mA cm^−2^ [87,88]. Cell viability gradually increased with experimental time, illustrating the sluggish deprivation of the MEA. After 120 h of the experiment, the cell voltage was improved from the initial 2.15 V to 2.35 V, and a decay rate of 1.61 mV h^−1^ was determined according to the slope of the fitted line. The stability of the cell with PTP-90 AEM showed analogous deprivation performance under similar experimental conditions, with the cell voltage progressively improving with an initial 40 h test time and a sharp increase to 2.8 V up to 120 h. 

In summary, AEMs are not chemically stable when exposed to alkaline environments, and one of the major obstacles they face in commercial usage is their implementation in alkaline environments. In addition, the technology of AEM electrolysis is still in its infancy. This mini-review article provides insights on the recently reported anion exchange membrane-based water electrolyzer and its operating performances. Table 3 shows the different AEMs and their preliminary characteristics in AEMWE operating conditions. 

## 3. Conclusions and Future Perceptions

Despite the substantial benefits and noteworthy progress of AEMWE, the realization of pilot-scale H_2_ creation by AEMWE is still in a burgeoning stage, with numerous obstacles to overcome for industrial operations [89]. A major challenge of AEMWE is poor cell behavior, particularly stability. Thus, the following problems need to be rectified for real-world AEMWE applications.

(i) Membranes: The growth of anion exchange membrane products must preserve several characteristics, namely anion exchange capacity, durability, anionic conductivity, water uptake, and swelling ratio. Basic studies must be carried out to determine in what way the deprivation of the anion exchange membrane occurs in a specific working atmosphere and to engineer supporting constituents. Using in situ examination and calculation tactics can be useful in scheming optimal membranes and understanding the operational mechanisms. Further, the progress of AEMs is important in several dimensions such as molecular design, phase manipulation, electrochemical properties, and application in AEMWEs. Several plausible future trends in AEM design emerge. (A) For cations, densely strung clusters and alkyl substituents are diffused to ensure OH^−^ ion conductivity and alkaline stability, respectively; N-cyclic quaternary ammoniums may direct a new era of enhanced anion exchange membrane behavior. (B) Ether-free polyaromatic supports with reinforcement are now promising for AEM stability necessities; hydrophilic/hydrophobic alterations are dependable for microphase separation to make transport highways; a novel network topology understanding develops as a potential method for AEM.
  Attaining the high performance of AEM material is still in the early stages and will be followed by further development of large-scale processing and low-cost fabrication to satisfy the part of universal energy.
(ii) Catalysts: Effective OER and HER electrocatalysts have been explored in both mathematical calculations and laboratory methodologies. However, based on the working environment of the AEMWE, the essential electrocatalyst resources vary. The AEMWE behaviour is assessed in aqueous potassium hydroxide, carbonate/bicarbonate, and water, and plenty of studies have described potassium hydroxide-fed conditions, where the platinum group of metal-free catalysts shows outstanding electrocatalytic characteristics. Nevertheless, the AEM water electrolyzer must be functioned in a clean water-fed environment eventually. The obstacles stem from the fact that the performance of the most extensively studied nickel catalysts degrades considerably below pH 9. The expansion of durable electrocatalysts in the clean water-fed process is unavoidable.
(iii) Operating conditions: The AEMWE operation is essential. Although the membranes and electrocatalysts have exceptional characteristics, AEMWE performance is lower than the expected outcomes when constructing membrane electrode assemblies or gathering the modules owing to the weight allocation losses, electrochemical resistances, and so on. Further, based on the device’s operating environment, the behavior of an AEM water electrolyzer differs, indicating that the appropriate working environment must be verified to attain higher current densities and long-lasting durability. When executing a stack cell beyond a single unit cell, the performance of electrolytes must be deliberated since the laminar flow or turbulence disturbs the performance of the stack cell.
(iv) It is difficult to quantitatively match the behaviour of anion exchange membranes or AEMWEs because the operational conditions or the determining techniques greatly affect the properties. In the event of the prevailing water electrolysis half-cells, the catalysts or the electrode characteristics could be matched with the Tafel slope, overpotential, and so on. It is well established, and it is likely to originate in an analogous working condition. Nevertheless, even with the standardized measurement methods, it is hard to replicate the characteristics of the AEM in real-world working environments. There are discrepancies between the reported characteristics of the marketable MEAs and the results attained from the test center. The electrolysis behaviors of AEMWEs are often disturbed by the working temperature, feed solution, etc. Thus, it is imperative to develop appropriate characterization methods that reflect the actual operating conditions and improve precise standards to assess the efficacy of AEMWEs.
(v) Durability: Regarding stability, accomplishing robust stability of AEMWEs appears to be less challenging than for AMFCs, though there are a few stability-limiting aspects depending on the device’s working conditions. However, it is significant to note that if AEM water electrolysis exchanges with PEM water electrolysis and alkaline water electrolysis methodologies, MEAs of AEMWE must have all the similar behavior and stability necessities. At present, AEMWEs do not have all the necessities, requiring essential development in particular zones before being employed in products. Though it is difficult to tell what working conditions of AEMWEs will be most advantageous for performance, price, stability, and method difficulty, growing research demands aimed at the enhanced behavior and stability of AEMWEs keep this technology encouraging and cautiously feasible. Ultimately, it will reach its own place in the quickly growing H_2_ economy.
(vi) Feeding solutions: The commonly employed feeding solution is an aqueous hydroxide solution owing to its enhancement of the anionic conductivity of AEMWEs and its consequent performance. Nevertheless, bearing in mind the lack of fresh water, the anticipated choices are seawater or treated wastewater. The above choices represent a challenging situation since AEMWE constituents rapidly worsen with pollutants existing in contaminated waters, and/or the behavior is lower due to modest reactions taking place in the electrodes. Further efforts in making AEMWE systems more robust and less vulnerable to feed-water contaminants are compulsory to enhance their durability and minimize their overall cost.

As a whole, the progress of extremely stable membranes and electrocatalysts should be pursued with priority. The formation of AEMs with suitable cations and main skeletons, which hinder the efficient activity of AEMWEs, and PGM-free catalysts with durability in appropriate electrolytes [90,91], requires thoughtful tactics toward MEA construction to diminish the efficiency losses. Finally, all modules must be optimized to minimize the methodical losses. With the help of basic research on MEAs, electrocatalysts, and membranes, AEMWE can become an established leader in H_2_ formation. Furthermore, during the electrolysis process altering the source of water from ultrapure to seawater, an optimal catalyst should be established because the local alterations in pH near the electrode are accelerated in seawater, leading to prominent catalyst degradation [92]. Furthermore, chloride ions in seawater can contribute to chloride oxidation reactions below high anodic potential, thus being more appealing than chlorine evolution reactions for suitable electrocatalysts with extraordinary selectivity towards OER to further develop the anion exchange membrane technology for pilot-scale H_2_ production [93].

## Figures and Tables

**Table 1:** Comparative illustration of the practical constraints for AWE, PEMWE, and AEMWE.

Parameters	AWE	PEMWE	AEMWE
Separation	Diaphragm	PEM	AEM
Cathode	Nickel molybdenum alloys	Pt group metals	Transition metals
Anode	Nickel cobalt alloys	RuO_x_, IrO_x_	Transition metals
Current collector plate	Nickel	Copper	Copper
Bipolar plate	---	Graphite and Ti	Ni or SS
Electrolyte	KOH	Pure water	Alkali solution, pure water
Current density	<0.5 A cm^−2^	1~2 A cm^−2^	1~2 A cm^−2^
Operating temperature	60–90 °C	50–90 °C	40–80 °C
Gas purity	>99.5%	>99.99%	>99.99%
Lifetime	~100 kh	<10 kh	<2 kh
Estimated cost	Low	High	---
Technology status	Mature	Commercial for small scale	Research and Development

**Table 2:** Merits and demerits of various WE techniques.

Electrolysis Methods	Merits	Demerits
AWE	➢ Well matured technology ➢ Non-noble electrocatalyst required ➢ Inexpensive technology ➢ High energy efficacy (70–80%) ➢ Materialized	➢ Poor current densities ➢ Inferior electrolyzer performance due to the formation of carbonates onto the surface of the electrode ➢ Purity of gases is low ➢ Low working pressure (3–30 bar) ➢ Poor vibrant behavior ➢ Large system setup required and poor stability
PEMWE	➢ Higher current density ➢ Comfortable arrangement ➢ Fast response ➢ Superior H_2_ formation rate with extraordinary purity of gases (99.99%) ➢ High energy efficacy (80–90%) ➢ High vibrant action	➢ Novel and moderately developed ➢ High price of the constituents ➢ Acidic atmosphere ➢ Noble metal catalysts required ➢ Poor stability ➢ Market stage is in near term
AEMWE	➢ Producing high-purity hydrogen ➢ Non-noble electrocatalyst required	➢ Low durability ➢ Research and development stage
SOE	➢ High efficacy (90–100%) ➢ Non-noble electrocatalysts required ➢ High operating pressure	➢ Research laboratory period ➢ Large system setup ➢ Poor stability

**Table 3:** Comparative illustration of the different AEMs in AEMWE cell performance and their characteristics.

Membrane	Anode	Cathode	Electrolyte	Temperature (°C)	Current Density	Durability (h)	Ref.
Quaternized poly [9,9-bis(6-bromohexyl) fluorene]-co [4,4-bis((4-phenyl) propyl) biphenyl)]	IrO_2_	Pt/C	1 M KOH	70	1531 mA cm^−2^	1000 h, 80 °C	[68]
Polysulfone-*g*-tetramethyl ammonium	Nickel foam	Pt/C	1 M KOH	90	1.3 A cm^−2^	---	[69]
Quaternized poly-carbazole	IrO_2_	Pt/C	1 M KOH	50	3.5 A cm^−2^	---	[70]
Cross-linked polybenzimidazole with norborene	IrO_2_	Pt/C	1 M KOH	60	150 mA cm^−2^	8.3 h	[71]
Piperidinium-*f*-poly(vinyl benzyl chloride) cross-linked by polybenzimidazole	Ir black	Pt/C	1 M KOH	60	2 V @ 700 mA cm^−2^	200 h 1.9 V (500 mA cm^−2^)	[72]
Piperidinium-*f*-styrene-ethylene-butylene-styrene copolymer	Ir-Black	Pt/C	Ultra-pure water 0.1 M KOH	60	2 V @ 275 mA cm^−2^ 2V @ 680 mA cm^−2^	300 h	[73]
Composite AEM (Celgard/Fumion)	Stainless steel	Stainless steel	0.5 M KOH	25	560 mA cm^−2^	163 h	[74]
N3-butyl Imidazolium blended with poly(vinyl alcohol)	IrO_2_	Pt/C	0.5 M KOH	60	2 V @ 547.7 mA cm^−2^	80 h (1.8 V)	[75]
Morpholinium-modified, polyketone-based AEM	Nickel foam	Nickel foam	Pure water	25	2.5 V @ 60 mA cm^−2^	---	[81]
Poly(fluorenyl-co-aryl piperidinium)	IrO_2_	Pt/C	1 M KOH	80	7.68 A cm^−2^ @ 2 V	1100 h, 0.5 A cm^−2^, 60 °C, 200 µV h^−1^	[82]
Poly(terphenyl piperidinium) based AEM	IrO_2_	Pt-Ru/C	0.1 M KOH	60	1.77 V @ 2.5 A cm^−2^	300 h, 1 A cm^−2^	[83]
Poly(biphenyl piperidinium)	Nickel foam	Pt/C	1 M KOH	60	2.2 V @ 2.8 A cm^−2^	---	[84]
Twisted ether-free poly arylene piperidinium	IrO_2_	Pt/C	5.6 wt.% KOH	50	200 mA cm^−2^	500 h (2.1 V)	[85]
Quaternized poly (terphenyl piperidinium) co (oxindole terphenylylene)	IrO_2_	Pt black	1 M NaOH	55	2.2 V @ 910 mA cm^−2^	400 mA cm^−2^, 120 h	[86]

## Data Availability

Not applicable.
